# Present State of Panel Practice

**Published:** 1913-11-01

**Authors:** 


### "Present State of Panel Practice": A Reply.

*To the Editor of* The Hospital.

Sir,—If any facts were needed to explain the present movement towards non-panel federation they are supplied by a consideration of the letter of "Northerner" in The Hospital of October 25. Some of his statements demand criticism. He says that the present inadequate panel service should be adjusted by the application of compulsion to all those practitioners who for good and conscientious reasons have declined voluntary service. One must at once point out that this is a preposterous proposal, for the simple reason that there is no precedent in any free country for coercing an unwilling worker even in the lowest ranks of labour, much less in the case of a free and liberal profession. Not even in such an important branch as that of the State defence, in which if anywhere it might be justifiable, have we yet arrived at compulsory service. The mere suggestion of such a thing coming from a medical man shows to what depths of degradation we have already sunk. So that when "Northerner" says "Every general practitioner should be bound to undertake so many panel patients" he is suggesting a remedy as impracticable as it is desperate.

And there are other reasons why it is impracticable. The conditions of compulsory insurance are, as he is well aware, applied to some fourteen millions of people without taking into consideration their topographical distribution. The insured persons are so located that in a typical large industrial town, including both crowded and residential areas, there is a greater number for Dr. A., who is on the panel and resides in a thickly populated district, than he is justified in undertaking. On the other hand, "Northerner" would compel Dr. B., conducting his own private practice in a more salubrious and distant area, to sacrifice his leisure or neglect his private work in order to accommodate the local Insurance Committee. However willing he might be to do so, the exigencies of time and space make it impossible. And this topographical difficulty applies with even greater force to thinly populated tracts, such as the Highlands, where at the moment the authorities are confronted with a deadlock; for we must remember that it has been officially stated that compulsory insurance has been applied to members of nine-tenths of the households of the kingdom.

There are also ethical objections to be met from the point of view of the insured person, who will not and certainly should not be coerced in the matter of his choice of doctor. In a large number of cases he has already indicated his resentment by sacrificing the voluntary contribution and paying the fees of a private doctor. The friendly societies in their recent conference this autumn spoke with no uncertain voice when their delegates, representing seven millions of insured persons, declared unanimously that they were dissatisfied with the quality of the medical service, which they described as inferior to that received prior to the passing of the Insurance Act (see *The Times*, September 19). There is no doubt, also, if one may judge from the dissatisfaction freely expressed on all sides, that the persons insured in the approved societies, representing over five millions, have come to a similar conclusion.

It is sometimes said that the remedy for the present state of things is not to be found in unfavourable criticism from those who object to the Act. At the same time the present duty of the medical profession is not to rectify faulty legislation, but rather to decline to be exploited for the convenience of any political party. Its immediate function should be to retrace the faulty steps and make a determined effort to retain that freedom of professional relations between it and the public so essential to the well-being of both.—I am, Sir, yours faithfully, Southerner.

October 25, 1913.

